# Response to comments on ‘Tuberculosis Outcomes among International Migrants in Europe: A Systematic Review and Meta-analysis’

**DOI:** 10.1016/j.ijregi.2025.100655

**Published:** 2025-04-26

**Authors:** Sergio Cotugno, Giacomo Guido, Nicola Veronese, Annalisa Saracino, Francesco Di Gennaro

**Affiliations:** 1 Clinic of Infectious Diseases, Department of Precision and Regenerative Medicine and Ionian Area (DiMePRe-J), University of Bari "Aldo Moro", Bari, Italy; 2 Saint Camillus International University of Health Sciences, Rome, Italy

**Keywords:** Tuberculosis, Migrants, Outcomes, Europe, Non-migrants

We thank Estaji et al. [1] for their insightful remarks on our article [2]. They rightly highlight several crucial issues that are central to the ongoing research agenda on migration and tuberculosis (TB), and we appreciate the opportunity to elaborate on them.

First, we acknowledge the relevance of the TB case definition. However, the diagnostic work-up for TB is currently undergoing important revisions, with new challenges emerging due to the increasing recognition of subclinical forms, the role of molecular tools, and presumptive TB. For this reason, we chose not to restrict our inclusion criteria to a specific diagnostic modality. Furthermore, it is worth noting that global TB burden estimates are largely based on case notifications, regardless of microbiological confirmation. Our approach aimed to reflect this reality and include the widest possible spectrum of real-world TB diagnoses [3].

Second, we agree that the term “migrant” encompasses a heterogeneous population, and oversimplification may neglect the evaluation of some aspects. However, it is also important to recognize that migrant status is a dynamic and non-linear process. An individual may begin as an internally displaced person, become an asylum seeker, then a labor migrant, and eventually acquire long-term residence status—or conversely face deterioration in living conditions and legal status. Moreover, social determinants such as language barriers, undocumented status, and precarious access to healthcare often cut across these categories [4]. Migration trajectories and the shared living conditions in host countries frequently create multicultural and multinational contexts where geographic origin, though still relevant, is only one of several layers influencing TB risk.

Third, we understand and share the concern regarding adjustment for confounding factors. Due to the design of the included studies—most of which were not specifically conceived to compare migrant vs non-migrant outcomes—we were unable to extract pooled data on key confounders such as comorbidities and socioeconomic indicators. Nevertheless, we strongly agree that future research should focus on these factors to better elucidate causal pathways and disease trajectories, as suggested by Estaji et al. [1]. This will be essential for generating evidence that is both rigorous and policy-relevant.

In conclusion, we fully endorse the need for more granular, disaggregated, and prospective research to inform effective and equitable TB control strategies. Without a thorough understanding of the social determinants and structural barriers affecting migrant populations, public health interventions risk being either too generic or poorly adapted to the realities on the ground.

## Declarations of competing interest

The authors have no competing interests to declare.
